# Exploration of Immune-Related Gene Expression in Osteosarcoma and Association With Outcomes

**DOI:** 10.1001/jamanetworkopen.2021.19132

**Published:** 2021-08-03

**Authors:** Wangmi Liu, Xiankuan Xie, Yiying Qi, Jiayan Wu

**Affiliations:** 1 Second Affiliated Hospital, College of Medicine, Zhejiang University, Hangzhou, China; 2 Shanghai Tenth People’s Hospital, Tongji University School of Medicine, Shanghai, China

## Abstract

**Importance:**

Host immune dysregulation is associated with initiation and development of osteosarcoma. In addition, immunotherapy for osteosarcomas requires some knowledge of the immune state of patients.

**Objective:**

To perform an immunogenomic landscape analysis based on The Cancer Genome Atlas (TCGA) project, which provides osteosarcoma samples with clinical information.

**Design, Setting, and Participants:**

This genetic association study was conducted from July 20, 2020, to September 20, 2020, as a secondary analysis of public data. Cox regression and risk score analyses were used to construct signatures of immune-related genes (IRGs) in 84 patients with osteosarcoma from TCGA with corresponding clinical information. Patients were divided into high- and low-risk groups with 42 individuals in each group according to their risk scores. Data were analyzed from July 20 to September 20, 2020.

**Main Outcomes and Measures:**

Differentially expressed genes (DEGs) were analyzed between groups, and potential molecular mechanisms, expression regulation, and immune cell infiltration were also explored using bioinformation methods. A prognostic model based on independent risk factors selected from multivariate Cox hazard ratio regression was established to estimate 1-year overall survival.

**Results:**

In this genetic association study based on 84 samples from patients with osteosarcoma from TCGA (mean [SD] age, 15.0 [4.8] years; 47 [56.0%] men; mean [SD] follow-up time, 4.1 [2.8] years), a total of 14 survival-associated IRGs were identified. Patients assigned to the high-risk group had worse survival than patients from the low-risk group (1 death [2.4%] vs 26 deaths [61.9%%]; *P* < .001). The protein digestion and absorption pathway was one of the associated pathways in the functional enrichment analysis (gene ratio, 2:8; *P* < .001). The prognostic model based on metastases at diagnosis and risk score performed well in 1-year overall survival estimations (area under the curve, 0.947; 95% CI, 0.832-0.972). The risk score was correlated with immune cell infiltration (B cells: *r* = 0.331; *P* = .002; macrophages: *r* = 0.410; *P* < .001; CD8 T cells: *r* = 0.230; *P* = .04).

**Conclusions and Relevance:**

This genetic association study developed a prognostic modeling tool for osteosarcoma based on IRG expression profiles, which could result in improved survival rates through more individualized therapies. Further research on IRG expression profiles could provide potential targets for future studies on immune treatment for osteosarcoma.

## Key Points

**Question:**

What is the immunogenomic landscape of osteosarcoma?

**Findings:**

In this genetic association study based on 84 samples from The Cancer Genome Atlas, 14 immune-related genes associated with survival in osteosarcoma were identified.

**Meaning:**

These findings suggest that a diagnostic risk score based on immune-related gene expression profiles may be useful to planning individualized therapies for osteosarcoma.

## Teaser

This genetic association study examines immune-related gene expression in osteosarcoma samples from The Cancer Genome Atlas project and assesses the association of gene expression profiles with survival outcomes.

## Introduction

Osteosarcoma is the most common primary malignant bone tumor, and tends to occur in young people. Distal femur (43%), proximal tibia (23%), and humerus (10%) are the most common sites.^1^ Significant pain and swelling of affected bones are essential characteristics, and osteosarcoma can cause pathological fractures in some cases. The overall survival (OS) rates are 67% after 2 years, 49% after 5 years, and 42% after 10 years.^2^ It is noteworthy that 15% to 20% of patients have metastases at diagnosis, and the OS of these patients is poor.^2,3^ The lung is the most common site of metastases, followed by bone. For newly diagnosed osteosarcoma, it is difficult to differentiate between patients with high or low risk at presentation. Therefore, it is necessary to develop new prognostic biomarkers that also can be used as alternative individual therapeutic targets.

The treatment for osteosarcoma has advanced from amputation to limb-sparing surgery with implants. Recently, immunotherapy, such as adoptive cellular therapy, vaccination, and checkpoint inhibitors, has been becoming an attractive therapeutic strategy.^4,5^ Preclinical work has shown encouraging results of anti–programmed cell death 1 and anti–programmed cell death 1 ligand 1 blockade therapy in both an osteosarcoma model in humanized mice and the lung metastases of osteosarcoma.^6,7^ However, in a 2017 randomized clinical trial by Tawbi et al,^8^ only 1 of 22 patients (5%) with osteosarcoma had an objective response to pembrolizumab, an anti–programmed cell death 1 antibody. The human immune system, including innate and adaptive immunity and immunocytes, plays a critical role in the tumor microenvironment. Different immunocytes have different features and performance characteristics. For example, neutrocytes inhibit natural killer cell function, which leads to extravasation of tumor cells.^9^ Therefore, immunotherapy relies on the antitumor immunocompetence of immunocytes. A full understanding of the immune state of patients, such as immune-related gene (IRG) expression and immunocyte infiltration, would be useful for the successful implementation of immune therapy.

The aim of this study is to understand the possibility of IRGs as biomarkers associated with risk stratification in osteosarcoma. Differentially expressed genes (DEGs) were analyzed between the high- and low-risk groups. Computational analysis was conducted to explore molecular mechanisms, expression regulation and immune cell infiltration involved. This study was intended to provide immunogenomic landscape of osteosarcoma, and position survival-associated IRGs as candidates for clinical biomarkers and possible intervention points for anticancer therapy.

## Methods

This genetic association study was approved by the institutional ethics committee of the Second Affiliated Hospital of Zhejiang University, Hangzhou, Zhejiang Province, China. Per institutional policy, informed consent was waived because we did not collect any specimens from patients. The study protocol and reporting were developed according to the Strengthening the Reporting of Genetic Association Studies (STREGA) reporting guideline.

### Clinical Samples and Data Acquisition

We downloaded transcriptome RNA-sequencing data of osteosarcoma samples from the TCGA database.^10^ In total, 84 patients with complete clinical information (ie, age, sex, primary tumor site, metastatic state at diagnosis, survival time, and survival state) were included in our analysis. Follow-up was started at the time of diagnosis, and OS time was censored at the last date the patient was known to be alive. The expression profiles were extracted from transcriptome RNA sequencing data of the osteosarcoma samples. Raw count data were processed into reads per kilobase of exon per million reads mapped for further analyses. The Immunology Database and Analysis Portal (ImmPort) database provides IRG availability to users, which facilitates immunology research owing to its transparency and reproducibility.^11^ The Cistrome Cancer database serves as a comprehensive resource for transcription factor (TF) targets and enhancer profiles. It is an important resource for users to query TFs that are related to specific gene of interest.^12^

### Risk Stratification Analysis

First, we explored whether there might be IRGs associated with survival in osteosarcoma. The expression level of each IRG was assessed by univariate Cox regression analysis using the survival package in R software version 3.6.1 (R Project for Statistical Computing), and the top 30 IRGs with *P* < .005 were selected as candidate genes. These genes were incorporated in the stepwise multivariate Cox hazard ratio (HR) regression to finish further selection. The sum of multiplication of survival-associated IRGs expression level and corresponding coefficient was defined as the risk score. The median value of the risk score was set as the threshold value to group patients, and there were 42 patients in the high-risk group (≥median) and 42 patients in the low-risk group (<median).

### DEGs and Enrichment Analysis

DEGs between the high- and low-risk groups were analyzed using R software with a log_2_ | fold change | >1 and a false discovery rate less than 0.05 as the cutoff values. To explore potential molecular mechanisms of the DEGs, the list of DEGs was analyzed using the Database for Annotation, Visualization and Integrated Discovery^13^ to acquire gene annotation in terms of molecular functions, cell components, and biological processes. In addition, a graph of pathways in which DEGs may be involved was generated using the Kyoto Encyclopedia of Genes and Genomes (KEGG) pathways program.^14^

### TF-IRG Network Construction

TFs can regulate the expression of related genes by activation or inhibition. The differentially expressed TFs were identified from DEGs according to the TFs list from the Cistrome Cancer database. *P* < .001 and a correlation score greater than 0.4 were set as the cutoff values in the correlation analysis between survival-associated IRGs and TFs. The TF-IRG network was constructed and visualized using Cytoscape software version 3.7.2 (National Resource for Network Biology).^15^

### Prognostic Model Construction

Univariate and multivariate Cox HR regression based on the risk score and clinical factors (ie, age, sex, metastases, and primary site) were performed to identify independent risk factors associated with survival outcomes. The area under the receiver operating characteristic curve (AUC) was calculated via the survivalROC package in R to validate the performance of the prognostic model based on the independent risk factors. The 95% CIs for AUCs were calculated using the nonparametric bootstrap via survAccuracyMeasures package. The calibration curve was plotted to assess the performance of the prognostic model as internal validation.

### Immune Cell Infiltration Analysis

The Tumor Immune Estimation Resource web server (X Shirley Liu Lab) was used to explore tumor immune cell infiltration in osteosarcoma.^16^ We used the estimation module to run our own data by setting the cancer type as sarcoma. The associations among 6 subtypes of tumor-infiltrating immunocytes (ie, B cells, CD4 T cells, CD8 T cells, macrophages, neutrophils, and dendritic cells) and the risk scores were calculated. *P* values were 2-sided, and *P* < .05 was set as the cutoff value in the analysis.

### Statistical Analysis

The statistical significance of differences between high- and low-risk groups was assessed using unpaired *t* tests for the continuous variable (age) and χ^2^ tests for dichotomous variables (sex, metastases at diagnosis, primary site). These analyses were performed using R software. Unless otherwise indicated, statistical significance was set at 2-sided *P* < .05 for statistical tests. Data were analyzed from July 20 to September 20, 2020.

## Results

### Identification of Survival-Associated IRGs in Patients

**Figure 1.**
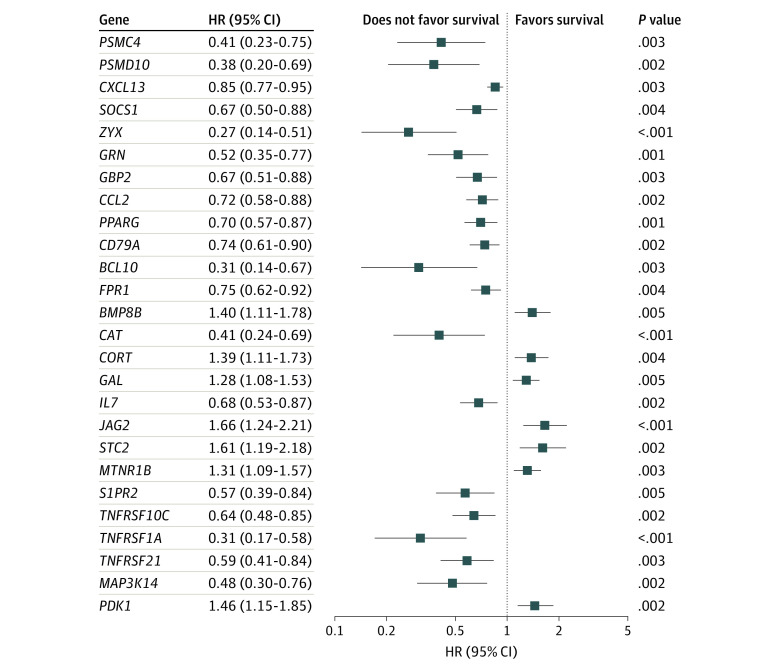
Immune-Related Genes Selected From Univariate Cox Regression Analysis HR indicates hazard ratio.

According to the defined criteria, RNA-sequencing data and clinical information from 84 patients (mean [SD] age, 15.0 [4.8] years; 47 [56.0%] men; mean [SD] follow-up time, 4.1 [2.8] years) were downloaded from the TCGA data portal. Since surveillance of disease outcomes is important for osteosarcoma management, we were committed to identifying survival-associated IRGs that could be used as viable prognostic indicators. In the univariate Cox regression analysis, we found 26 IRGs that were significantly correlated with OS in patients (Figure 1). In further analysis, 14 IRGs were identified as survival-associated IRGs using multivariate Cox regression analysis (Table).

**Table.** Multivariate Cox Regression Analysis of Survival Associated Immune-Related Genes

Gene	Coefficient	HR (95% CI)	*P* value
*PSMC4*	−1.239	0.29 (0.11 to 0.76)	.01
*CXCL13*	0.219	1.24 (0.98 to 1.59)	.08
*GBP2*	−0.650	0.52 (0.32 to 0.85)	.008
*CCL2*	0.507	1.66 (1.10 to 2.51)	.02
*PPARG*	−0.770	0.46 (0.31 to 0.70)	<.001
*CD79A*	−0.681	0.51 (0.34 to 0.77)	.001
*BCL10*	−1.525	0.22 (0.06 to 0.75)	.02
*FPR1*	−0.483	0.62 (0.45 to 0.85)	.003
*BMP8B*	0.481	1.62 (1.03 to 2.53)	.04
*CORT*	0.548	1.73 (1.23 to 2.44)	.002
*JAG2*	−0.735	0.48 (0.28 to 0.83)	.008
*STC2*	0.690	1.99 (1.26 to 3.16)	.003
*MTNR1B*	0.186	1.21 (0.93 to 1.58)	.17
*TNFRSF21*	−0.929	0.40 (0.20 to 0.77)	.007

Abbreviation: HR, hazard ratio.

### Risk Score–Based Patient Grouping

**Figure 2.**
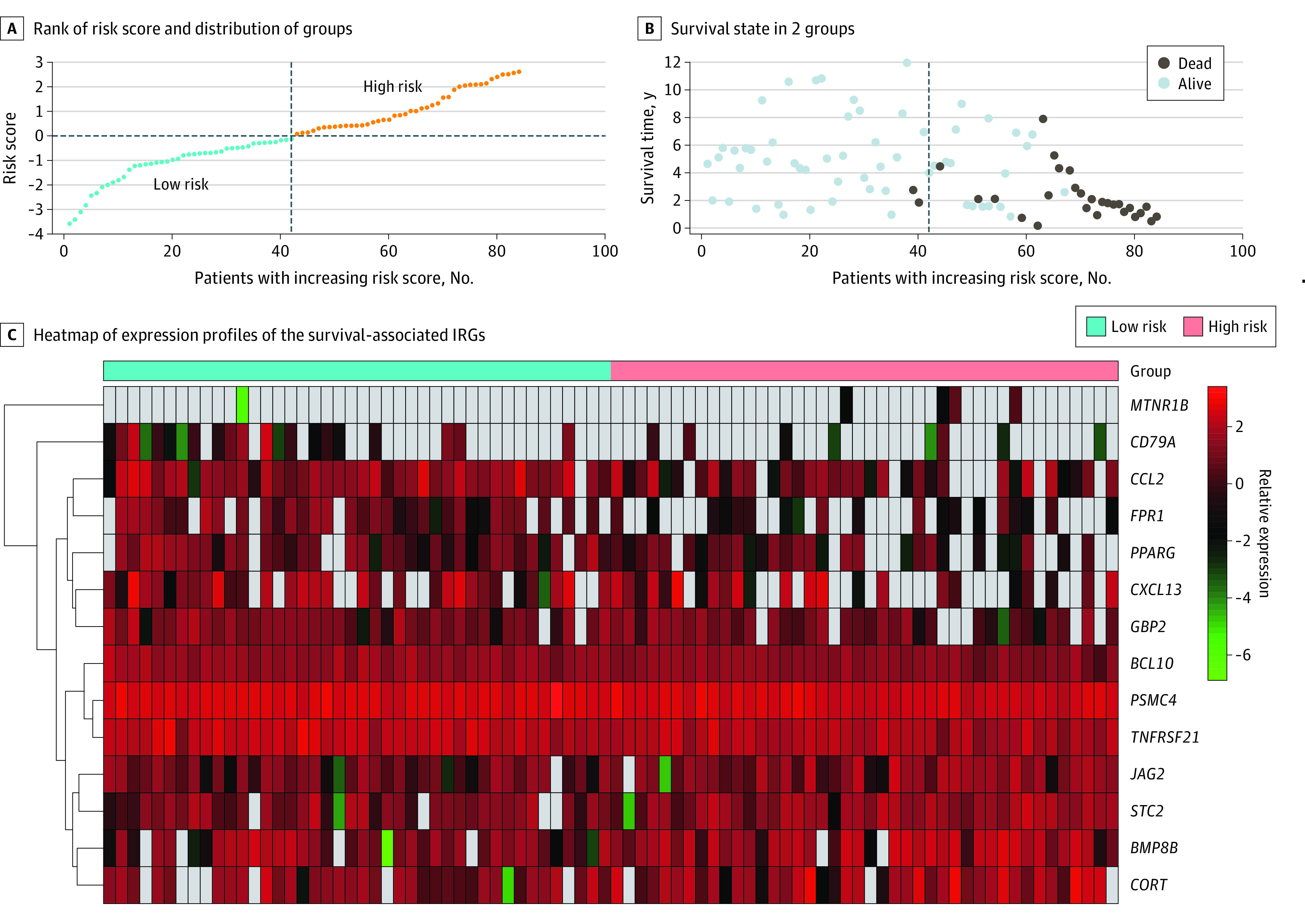
Clinical Outcomes Based on Risk Score IRG indicates immune-related gene.

According to the expression of survival-associated IRGs and their coefficients (Table), every patient received a risk score. A total of 42 patients with risk scores greater than or equal to the median value were grouped into the high-risk group, and 42 patients with risk scores less than the median value were grouped into the low-risk group. There was significant difference in metastases at diagnosis and survival state between groups (eTable 1 in the Supplement). Patients assigned to the high-risk group had worse survival than patients from the low-risk group (1 death [2.4%] vs 26 deaths [61.9%%]; *P* < .001). Survival analysis confirmed that patients in the high-risk group had much lower survival probability than those in the low-risk group, and the estimated 5-year survival rate was 30.6% (95% CI, 18.1%-51.9%) (eFigure 1 in the Supplement). Figure 2 shows the evaluation of clinical outcomes based on risk score.

### Identification of DEGs Between Groups

A total of 203 DEGs were obtained following data processing (eTable 2 in the Supplement). Compared with the low-risk group, 16 genes were significantly upregulated and 187 genes were significantly downregulated in the high-risk group (eFigure 2 in the Supplement). There were 3 survival-associated IRGs among the DEGs: *CCL2* (OMIM 158105), *CD79A* (OMIM 112205), and *FPR1* (OMIM 136537). They were all significantly downregulated in the high-risk group.

### Gene Ontology Enrichment Analysis

During analysis, DEGs were categorized into 3 major terms: cellular components, molecular functions, and biological processes. The significantly enriched gene ontology terms are presented in eTable 3 in the Supplement. The DEGs were most significantly associated with the inflammatory response, transmembrane signaling receptor activity, and the external side of the plasma membrane in the 3 categories.

### KEGG Pathway Enrichment Analysis

The upregulated DEGs were significantly associated with protein digestion and absorption (gene ratio, 2:8; *P* < .001), melanogenesis, and the sphingolipid signaling pathway. In contrast, the top 3 pathways correlated with the downregulated DEGs were *Staphylococcus aureus* infection (gene ratio, 12:103; *P* < .001), the hematopoietic cell lineage (gene ratio, 11:103; *P* < .001), and viral protein interactions with cytokines (gene ratio, 11:103; *P* < .001) (eFigure 3 in the Supplement).

### TF Regulatory Network

To explore the association between survival-associated IRGs and TFs, we first identified 3 TFs in the DEGs: CCAAT enhancer binding protein α (CEBPA), GATA binding protein 3 (GATA3), and TF 7. Compared with their expression in the low-risk group, the expression of CEBPA and GATA3 was lower in the high-risk group, while the expression of TF 7 was higher (eFigure 4A in the Supplement). We then constructed a TF-based regulatory network to show the associations and potential molecular mechanisms among these survival-associated IRGs (eFigure 4B in the Supplement). CEBPA was the core TF associated with regulating the expression of survival-associated IRGs.

### Establishment of the Prognostic Model

**Figure 3.**
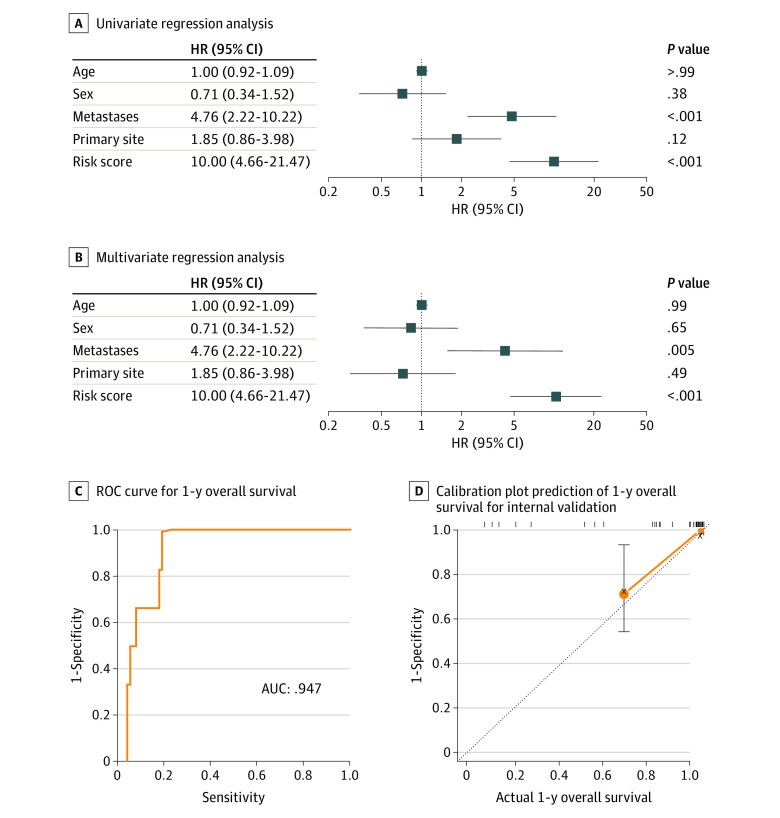
Estimation of 1-Year Overall Survival in Patients With Osteosarcoma AUC indicates area under the receiver operating curve; HR, hazard ratio.

Univariate regression analysis suggested that the prognostic factors included metastases at diagnosis and risk score (Figure 3A). The multivariate regression analysis confirmed that metastases at diagnosis and the risk score were independent risk factors after adjustments were made for age, sex, and primary site (Figure 3B). Thus, multivariate Cox regression based on metastases at diagnosis and risk score was performed to construct the prognostic model. Figure 3C shows that our prognostic model provided excellent prognostic performance (AUC, 0.947; 95% CI, 0.832-0.972). The calibration plot-estimated 1-year OS of osteosarcoma, built to internally validate prognostic performance based on metastases at diagnosis and risk score, performed very well compared with the ideal model (Figure 3D).

### Immunocyte Infiltration

**Figure 4.**
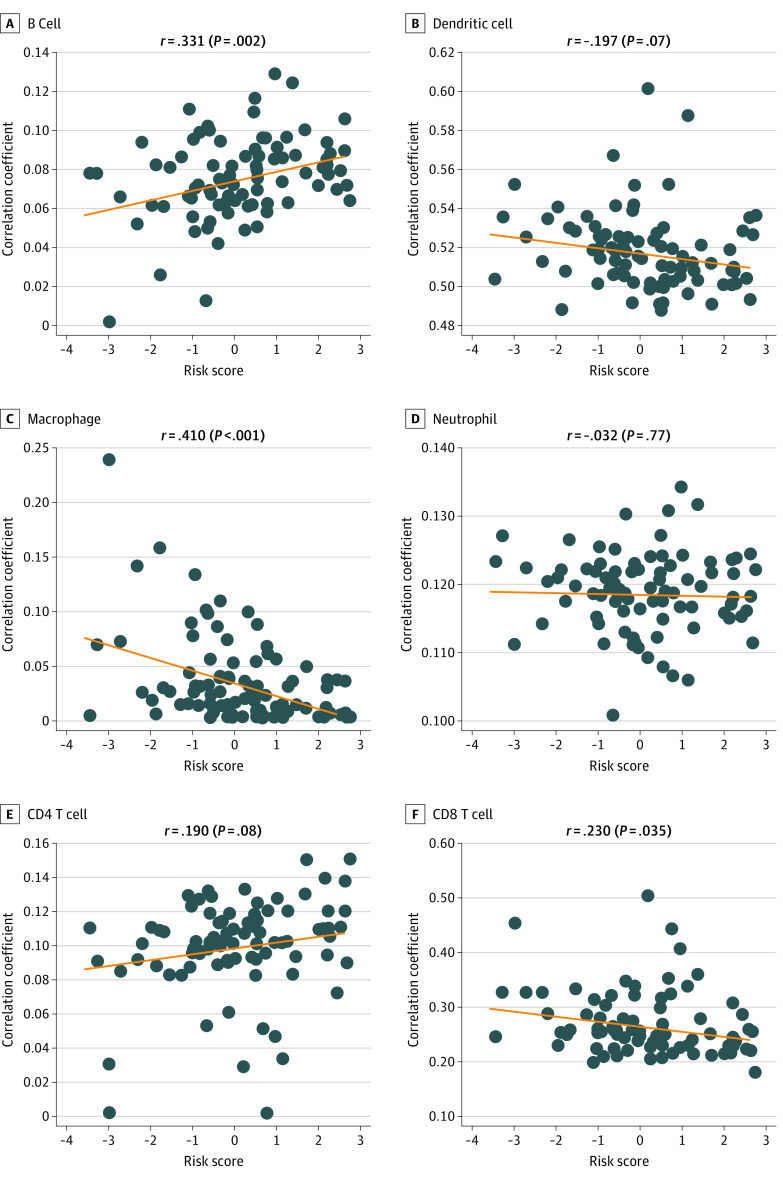
Immunocyte Infiltration Dots indicate individual data points; line, trend.

To decipher the immune microenvironment in osteosarcoma, we analyzed the association between immunocytes in osteosarcoma and risk score. B cell infiltration was positively correlated with the risk score (*r* = 0.331; *P* = .002), while macrophages (*r* = 0.410; *P* < .001) and CD8 T (*r* = 0.230; *P* = .04) cell infiltration were negatively correlated with risk score (Figure 4).

## Discussion

This genetic association study proposed a bioinformatics-based approach, including clinical data collection, transcriptome profiling, statistical analysis, determination of a regulatory network, and functional analysis, to decipher the potential molecular mechanisms of the multicomponent nature of the immune system and its role in osteosarcoma. Therefore, this approach provides a systemic perspective to study the immune microenvironment in osteosarcoma and can reveal the possible outcomes and mechanisms associated with survival-associated IRGs.

The risk score based on the IRGs had similar sensitivity and specificity to a classifier proposed by Liu et al.^17^ Of 14 survival-associated IRGs, 5 have been reported in studies about osteosarcoma, such as a 2018 study by Han et al.^18^ CCL2 is an important chemokine that plays a critical role in cancer progression. Previous studies have demonstrated that CCL2 promotes the proliferation and metastasis of osteosarcoma cells by activating the receptor activator of NF-κB ligand signaling pathway.^19,20^ CD79a is expressed exclusively on B cells. However, when patients with osteosarcoma receive high-intensity focused ultrasound ablation, there is no significant difference in the percentage of B cells before and after treatment.^21^ *FPR1* is expressed at high levels on neutrophils, monocytes, and macrophages. Formylated peptides can elicit production of reactive oxygen species, release of proteolytic enzymes, morphological polarization, and locomotion.^22^ A 2016 study by Vincenzo et al^23^ reported that the cyclized peptide SRSRY prevents key events during metastases via FPR1 on the cell surface. Jagged canonical Notch ligand 1 is markedly expressed in osteosarcoma cell lines. The Notch signaling pathway mediates the growth of osteosarcoma promoted by bone morphogenetic protein 9 (BMP9).^24,25^ Tumor necrosis factor receptor superfamily member 21 (TNFRSF21) overexpression induces cell death in osteosarcoma cells. Apoptosis induction in osteosarcoma by silencing of eukaryotic translation initiation factor 3 subunit B is mediated by upregulation of TNFRSF21.^26^ The other 9 survival-associated IRGs deduced from computational analysis may be potential targets for treatment. For example, like BMP9, bone morphogenetic protein 8b (BMP8b) is a member of the BMP family, and BMP9 and BMP8b may share some identical or similar outcomes in osteosarcoma. BMP9 has been shown to promote the proliferation of osteosarcoma cells,^24,25^ which is similar to this study’s finding that BMP8b was a risk factor in osteosarcoma. Therefore, the role of BMP8b in osteosarcoma needs further research.

The enriched gene ontology functions for the survival-associated IRGs were mainly related to the immune system, including immunocyte activation and immune response. The most significantly associated term in the biological processes category was inflammatory response. It has been reported that inflammatory response plays a critical role in tumor initiation, promotion, malignant conversion, invasion, and metastases.^27^ Inflammation also affects patient survival by regulating responses to therapy and immune surveillance.^28^ Specifically, research about the action of inflammation in promoting *KRAS*-driven oncogenesis has confirmed that inflammation collaborates with *KRAS* signaling to promote carcinogenesis.^29^ *KRAS* is a target of let-7a in osteosarcoma. However, a single-nucleotide variation (rs61764370) interferes with the interaction between the 3′-untranslated region of *KRAS* and let-7a, resulting in enhanced metastatic potential of osteosarcoma cells.^30^ In addition, exfoliated black phosphorus inhibits osteosarcoma progression via cancer-related–inflammation inhibition mechanisms, such as increased anti-inflammatory cytokine generation and decreased proinflammatory mediator synthesis.^31^ The main items in the molecular functions and cellular components categories were related to receptor activity and the membrane, respectively, which are consistent with the biological processes associated with the IRGs.

KEGG enrichment analyses showed that protein digestion and absorption were the most significantly upregulated pathways, which is similar to the findings of a 2017 study by Shi et al.^32^ For Wnt signaling pathway, there are inconsistencies in studies about osteosarcoma. Knockdown of homeobox B8 dramatically repressed the migration and invasion of osteosarcoma cells by preventing the activation of Wnt signaling pathway.^33^ A 2017 study^34^ has shown that Wnt signaling pathway activation may downregulate the expression of Beclin 1 and rescue chemotherapy drug resistance in osteosarcoma. This discrepancy could be ascribed to the complexity of Wnt signaling pathway. However, better knowledge about the Wnt signaling pathway is needed to safely target this pathway in osteosarcoma. Therefore, recent studies have proposed the implication of the Wnt signaling pathway in metastatic dissemination through angiogenesis and immune surveillance.^35^ Most of the downregulated pathways were related to immunocyte activation and immune response. It should be noted that the most downregulated pathway was *S aureus* infection. In the late 19th century, William B. Coley, MD, used bacterial immunotherapy to treat sarcomas.^36^ There is an association between improved survival in patients with osteosarcoma and infection.^37^ In vitro experiments have demonstrated that infection counteracted osteosarcoma-induced immune suppression through upregulating inflammatory immune response, as well as alterations in macrophage inflammatory profiles.^38^ This treatment provides a new strategy to improve antitumor immune responses.

In this study, CEBPA was the core TF in the TF-IRG regulatory network. CEBPA is a member of the CEBP family of TFs. Initially, CEBPA was characterized in adipogenesis. Later, researchers found its expression in multiple tissues as a tumor suppressor, including lung, liver, mammary glands, and skin. Mutation of CEBPA plays a pivotal role in leukemogenesis by converting hematopoietic stem cells to leukemia-initiating cells.^39^ In osteosarcoma, overexpression of *PLK2* impedes cell apoptosis and promotes cell proliferation exposed to endoplasmic reticulum stress. Recruiting CEBPA to the promoter of *PLK2* negatively regulates its expression.^40^ Therefore, like its role in acute myeloid leukemia, CEBPA is recognized as an antitumor TF in osteosarcoma. GATA3 also acts as a tumor suppressor and is involved in multiple types of cancer.^41,42^ GATA3 significantly inhibits the epithelial–mesenchymal transition-associated TF slug, which suppresses cell proliferation, migration, and invasion in osteosarcoma. Unfortunately, GATA3 is downregulated in patients with osteosarcoma.^43^ We also found that GATA3 was lower in the high-risk group than in the low-risk group.

The clinical application of cancer immunotherapy requires the study of the relationship between host immune system and tumor cells. However, the size and complexity of tumor-immune interactions hinder our ability to easily identify the associations between immunocyte infiltration and clinicopathological factors. In this study, we used the Tumor Immune Estimation Resource database to comprehensively investigate the association between immunocyte infiltration and our risk score.^16^ B cell infiltration was positively associated with risk score. Few studies have addressed B cell infiltration in osteosarcoma. In addition, existing data on the role of B cell infiltration are inconsistent. One mouse tumor model showed that B cell infiltration promoted tumor inflammation^44,45^ but also inhibited the antitumor T cell–dependent response.^46,47^ We identified that infiltration of CD8 T cells and macrophages was negatively associated with our risk score. Numerous macrophages, which can control the behavior of tumor cells, such as tumor cell migration and invasion, angiogenesis, and local immunity, invade osteosarcoma tissues.^48^ Therefore, the total number of macrophages is associated with osteosarcoma survival.^4^ T-lymphocytes are the second most infiltrating immunocyte in osteosarcoma tissues.^49^ In a 2015 study, Fritzsching et al^50^ observed improved estimated survival in patients with higher CD8^+^ to FOXP3^+^ ratios compared with survival in patients with lower ratios.

### Limitations

This study has some limitations. First, transcriptome analysis cannot reflect overall immune state changes. Second, the results from our bioinformatics analysis lack in vitro experiments and validation in an independent cohort of patients. Third, owing to the requirement of complete clinical information, the sample size was not large. Fourth, because the database provides limited clinical information, other important factors, such as staging and grading, were not included in our analysis. Therefore, extrapolation based on the findings must be done very carefully.

## Conclusions

This genetic association study systematically investigated IRGs in osteosarcoma based on their functions, associated pathways, regulatory network, efficacy levels, and clinical applications. Immune states are heterogeneous among patients even with the same tumor type and may impact the effect of clinical immunotherapy. Because of the complexity and plasticity in the tumor microenvironment, it remains a challenge to study the interactions between tumor cells and immunocytes. The survival-associated IRGs examined in this study have potential for identifying prognosis in osteosarcoma and may be clinically useful as relevant clinical biomarkers and candidate targets for anticancer therapy in future research.

## Notes

Supplement. **eTable 1.** Clinicopathological Characteristics of the Patients **eTable 2.** DEGs Between the High- and Low-Risk Groups **eTable 3.** Top 10 Most Significant Terms in GO Enrichment Analyses **eFigure 1.** Overall Survival According to the Risk Score in Patients With Osteosarcoma **eFigure 2.** Volcano Plot and Heatmap of DEGs Between Groups **eFigure 3.** KEGG Pathway Enrichment Analyses: the Enriched KEGG Signaling Pathways From the DEGs **eFigure 4.** TFs and IRGs Associations
