# Supplementary material for: Exploration of Immune-Related Gene Expression in Osteosarcoma and Association With Outcomes
Source: JAMA Netw Open. 2021 Aug 3;4(8):e2119132. doi: 10.1001/jamanetworkopen.2021.19132 (PMC8335580; doi:10.1001/jamanetworkopen.2021.19132)
Supplement: Supplement. — eTable 1. Clinicopathological Characteristics of the Patients eTable 2. DEGs Between the High- and Low-Risk Groups eTable 3. Top 10 Most Significant Terms in GO Enrichment Analyses eFigure 1. Overall Survival According to the Risk Score in Patients With Osteosarcoma eFigure 2. Volcano Plot and Heatmap of DEGs Between Groups eFigure 3. KEGG Pathway Enrichment Analyses: the Enriched KEGG Signaling Pathways From the DEGs eFigure 4. TFs and IRGs Associations [file jamanetwopen-e2119132-s001.pdf]

Supplemental Online Content

Liu W, Xie X, Qi Y, Wu J. Exploration of immune-related gene expression in osteosarcoma and association with outcomes. *JAMA Netw Open*. 2021;4(8):e2119132.
doi:10.1001/jamanetworkopen.2021.19132

eTable 1. Clinicopathological Characteristics of the Patients
eTable 2. DEGs Between the High- and Low-Risk Groups
eTable 3. Top 10 Most Significant Terms in GO Enrichment Analyses
eFigure 1. Overall Survival According to the Risk Score in Patients With Osteosarcoma
eFigure 2. Volcano Plot and Heatmap of DEGs Between Groups
eFigure 3. KEGG Pathway Enrichment Analyses: the Enriched KEGG Signaling Pathways From the DEGs
eFigure 4. TFs and IRGs Associations

This supplemental material has been provided by the authors to give readers additional information about their work.

© 2021 Liu W et al. *JAMA Network Open.*

eTable 1. Clinicopathological Characteristics of the Patients

**Variables**	**Low risk group, n=42**	**High risk group, n=42**	**P**
Age (years)	15.02±4.84	14.93±4.78	0.930
Gender			0.183
Female	15 (35.7%)	22 (52.4%)	
Male	27 (64.3%)	20 (47.6%)	
Metastases at diagnosis			0.011
Presence	5 (11.9%)	16 (38.1%)	
Absence	37 (88.1%)	26 (61.9%)	
Primary site			0.125
Femur	15 (35.7%)	23 (54.8%)	
Other site	27 (64.3%)	19 (45.2%)	
Survival state			
Live	41 (97.6%)	16 (38.1%)	
Dead	1 (2.4%)	26 (61.9%)	<0.001

© 2021 Liu W et al. *JAMA Network Open.*

eTable 2. DEGs Between the High- and Low-Risk Groups

Gene symbol	Low risk (Mean of log 2(RPKM))	High risk (Mean of log 2(RPKM))	logFC	P Value	FDR
MIR4768	3.981	-0.074	-4.055	0.0004128	0.032
CSAG1	2.259	-1.681	-3.940	0.0003805	0.031
MIR135A1	2.193	-1.740	-3.934	0.0001728	0.023
MAGEA3	3.153	-0.756	-3.909	0.0007723	0.042
PAGE5	2.843	-0.905	-3.747	0.0011018	0.047
MAGEA12	1.406	-2.233	-3.638	0.0006961	0.040
SSX1	2.749	-0.578	-3.327	0.0009114	0.044
LINC01522	-1.982	-5.229	-3.247	6.04E-05	0.016
LINC02104	1.136	-2.104	-3.240	3.52E-06	0.007
MAGEB2	-0.535	-3.652	-3.117	0.000583	0.037
MYL1	0.567	-2.463	-3.030	0.000234	0.027
MAGEA11	-0.880	-3.791	-2.911	0.0001176	0.020
STRA8	-0.443	-3.353	-2.910	3.29E-05	0.013
COX7B2	-1.658	-4.236	-2.579	0.0006707	0.039
MAGEC1	-1.992	-4.552	-2.560	0.0011169	0.047
LINC00626	2.055	-0.485	-2.540	0.0002908	0.029
XCL2	0.699	-1.821	-2.520	0.0001773	0.023
SMPX	-2.188	-4.645	-2.457	0.0006359	0.038
LINC02234	-2.431	-4.882	-2.452	0.0001499	0.023
IGLL5	-1.098	-3.523	-2.425	0.0007493	0.041
C5orf46	-1.638	-4.046	-2.408	0.0009228	0.044
XAGE5	-2.762	-5.169	-2.408	0.0011044	0.047
CCL8	1.747	-0.607	-2.354	1.72E-05	0.009
RSPO3	0.181	-2.117	-2.298	0.0002163	0.026
C11orf91	-2.093	-4.299	-2.207	8.58E-05	0.018
PF4	1.515	-0.633	-2.148	0.0006938	0.040
ACTG2	1.237	-0.870	-2.106	7.43E-05	0.017
MSC	4.074	2.005	-2.069	6.61E-06	0.008
FMO3	-0.191	-2.231	-2.040	5.21E-06	0.008
LINC01234	-2.195	-4.207	-2.012	0.0003111	0.029
MIR3945HG	-1.260	-3.260	-2.000	0.0006141	0.038
CXCL11	0.599	-1.386	-1.985	0.0005271	0.036
APOBEC3H	-1.504	-3.467	-1.962	0.0006154	0.038
LINC01444	-2.989	-4.950	-1.961	0.0012384	0.050
HGF	-0.116	-2.049	-1.932	8.36E-06	0.008
LARS2-AS1	-2.180	-4.095	-1.914	0.0009172	0.044
GJA5	1.809	-0.087	-1.896	2.90E-06	0.007
AGMO	-1.258	-3.144	-1.886	0.0001568	0.023
ST6GAL2	-0.340	-2.225	-1.884	0.0011073	0.047

© 2021 Liu W et al. *JAMA Network Open.*

Gene symbol	Low risk (Mean of log 2(RPKM))	High risk (Mean of log 2(RPKM))	logFC	P Value	FDR
IL2RA	-0.909	-2.793	-1.884	5.46E-06	0.008
GBP1P1	-0.835	-2.706	-1.872	0.0003952	0.031
VSIG4	3.942	2.077	-1.865	3.05E-06	0.007
FPR1	1.296	-0.550	-1.846	6.19E-07	0.007
LINC01678	-0.521	-2.352	-1.831	0.0001906	0.024
HRCT1	2.660	0.833	-1.827	0.0001778	0.023
VCAM1	2.478	0.653	-1.825	1.99E-05	0.009
TMIGD2	-2.132	-3.954	-1.822	0.0003461	0.030
MYOSLID	0.467	-1.336	-1.803	0.0003283	0.029
CD2	1.160	-0.624	-1.783	0.0001303	0.021
BBOX1	-0.931	-2.686	-1.755	0.0004024	0.031
SBSN	-2.440	-4.184	-1.744	0.000556	0.036
LINC02262	-3.772	-5.509	-1.737	0.0009221	0.044
CPA4	-2.075	-3.809	-1.734	0.0010052	0.046
CCL2	2.980	1.263	-1.716	3.65E-05	0.013
ACTN1-AS1	-0.119	-1.831	-1.711	0.0003225	0.029
LURAP1L	1.297	-0.407	-1.704	3.81E-05	0.013
C1orf194	-1.505	-3.203	-1.698	0.0006416	0.038
LINC00525	-2.545	-4.213	-1.667	0.00053	0.036
C1QA	7.753	6.095	-1.658	3.81E-05	0.013
C1QC	8.135	6.485	-1.651	1.21E-05	0.008
JPH2	-0.371	-2.022	-1.651	1.01E-05	0.008
F13A1	2.985	1.337	-1.648	9.11E-05	0.018
ACTA2	5.565	3.919	-1.646	4.15E-05	0.013
C1QB	7.203	5.573	-1.630	2.27E-05	0.010
GZMB	1.183	-0.438	-1.621	0.0010425	0.046
CD3E	0.421	-1.189	-1.611	0.0001646	0.023
MMP8	-2.627	-4.235	-1.608	0.0003437	0.030
SIGLEC14	0.524	-1.083	-1.607	0.0002278	0.027
IL33	-2.141	-3.745	-1.604	0.0011681	0.048
SIT1	0.050	-1.548	-1.598	2.32E-05	0.010
BBOX1-AS1	0.455	-1.129	-1.585	0.0010593	0.046
CCIN	-1.731	-3.315	-1.584	9.38E-05	0.018
CTF1	0.908	-0.671	-1.579	0.0003247	0.029
FMO2	-2.921	-4.500	-1.579	0.0004919	0.035
P2RY10	-2.160	-3.739	-1.579	0.0004294	0.032
KLRB1	0.200	-1.373	-1.573	0.0003172	0.029
GBP1	3.219	1.658	-1.561	1.01E-05	0.008
IL10	-0.641	-2.200	-1.559	9.17E-06	0.008
NCF1B	0.179	-1.374	-1.553	2.47E-05	0.010

© 2021 Liu W et al. *JAMA Network Open.*

Gene symbol	Low risk (Mean of log 2(RPKM))	High risk (Mean of log 2(RPKM))	logFC	P Value	FDR
TTC9B	-1.539	-3.089	-1.550	1.05E-05	0.008
CD79A	0.055	-1.479	-1.533	0.0006017	0.038
HCST	4.132	2.614	-1.518	0.0009362	0.045
CD5	-1.081	-2.597	-1.516	7.82E-05	0.017
TNFSF8	0.153	-1.358	-1.511	4.29E-06	0.008
CXCL12	2.317	0.814	-1.503	4.52E-05	0.013
CD27	0.423	-1.079	-1.502	0.0001461	0.022
GATA3	-0.249	-1.738	-1.489	6.85E-05	0.017
PCDHB6	-0.008	-1.494	-1.486	0.0006196	0.038
DCAF8L1	-4.717	-6.194	-1.477	0.0001109	0.020
TAGLN	4.365	2.892	-1.473	3.53E-06	0.007
STAP1	-2.425	-3.883	-1.458	0.0011523	0.048
CD3G	-1.694	-3.146	-1.451	0.0003283	0.029
NKG7	2.845	1.406	-1.439	0.000905	0.044
VGLL3	-1.834	-3.267	-1.432	0.0011448	0.048
CD7	0.141	-1.284	-1.425	0.0003247	0.029
MS4A4A	2.698	1.285	-1.413	2.59E-05	0.010
CD52	3.450	2.037	-1.413	0.0005195	0.035
LY96	5.671	4.263	-1.408	0.000192	0.024
CD80	-1.287	-2.683	-1.396	0.0003764	0.030
TSTD1	0.582	-0.803	-1.386	0.0001465	0.022
CD163	2.900	1.537	-1.363	8.75E-05	0.018
FCGR3A	3.882	2.530	-1.352	0.0008171	0.043
FCER1G	7.379	6.031	-1.348	0.0005776	0.037
LINC02328	1.624	0.278	-1.346	0.0007896	0.042
CCDC42	-1.386	-2.728	-1.342	0.0009937	0.046
TREM2	4.756	3.420	-1.336	0.0003369	0.029
TMEM176B	4.512	3.187	-1.324	0.0001253	0.021
FCGR1A	1.310	-0.007	-1.317	0.0002153	0.026
TLR7	-0.356	-1.670	-1.315	0.000313	0.029
ISG15	7.854	6.545	-1.309	0.0012233	0.049
PRDM16	-3.220	-4.526	-1.305	0.0008337	0.043
CD8A	-0.501	-1.807	-1.305	0.0008748	0.044
P2RY6	0.128	-1.175	-1.303	8.07E-05	0.018
TYROBP	8.367	7.066	-1.301	0.000905	0.044
CD14	6.284	4.984	-1.300	0.0001584	0.023
LAX1	-2.595	-3.888	-1.294	0.0006318	0.038
C3AR1	3.603	2.311	-1.292	8.76E-06	0.008
GPR65	-0.031	-1.316	-1.285	1.21E-05	0.008
S1PR4	0.024	-1.261	-1.285	0.0008748	0.044

© 2021 Liu W et al. *JAMA Network Open.*

Gene symbol	Low risk (Mean of log 2(RPKM))	High risk (Mean of log 2(RPKM))	logFC	P Value	FDR
APOC1	3.093	1.816	-1.278	0.0002236	0.026
SERTAD4-AS1	2.038	0.762	-1.276	0.0007121	0.040
APOE	6.398	5.121	-1.276	0.0003759	0.030
CRYBB1	1.501	0.228	-1.273	0.0002411	0.027
GPR141	-2.063	-3.331	-1.268	0.000192	0.024
TMEM176A	2.122	0.869	-1.253	0.0006196	0.038
CYBB	3.352	2.100	-1.252	0.0001995	0.025
NCF1C	1.606	0.368	-1.238	0.000905	0.044
GPR34	2.966	1.728	-1.238	0.0003247	0.029
LY86	3.210	1.975	-1.235	0.000192	0.024
MNDA	2.297	1.079	-1.218	5.81E-05	0.015
TNFSF13B	1.662	0.446	-1.216	0.0001027	0.019
FZD2	3.162	1.947	-1.215	0.00028	0.029
SMIM25	0.075	-1.140	-1.215	0.0003247	0.029
ALDH1A1	1.846	0.633	-1.213	0.0007896	0.042
CYP2S1	0.403	-0.806	-1.210	4.91E-05	0.013
PTPRH	-1.692	-2.902	-1.210	0.0010101	0.046
SOWAHD	0.095	-1.111	-1.206	0.0007371	0.040
LRRC25	2.650	1.456	-1.194	8.40E-05	0.018
OLR1	-0.368	-1.562	-1.194	0.0011834	0.048
MRC1	1.667	0.481	-1.186	0.0001523	0.023
PCED1B	1.009	-0.170	-1.179	4.71E-05	0.013
TMEM150B	1.253	0.082	-1.171	0.0005013	0.035
PTGDR	-1.147	-2.317	-1.170	0.0006416	0.038
SECTM1	2.485	1.318	-1.167	0.0004504	0.033
TNFSF10	2.584	1.419	-1.165	0.0003016	0.029
FPR3	3.516	2.352	-1.164	0.000313	0.029
MS4A6A	2.191	1.029	-1.163	0.0004838	0.035
CASTOR1	-1.095	-2.247	-1.152	1.45E-05	0.008
PODN	1.818	0.667	-1.151	0.0007371	0.040
SASH3	2.201	1.051	-1.149	0.0001303	0.021
VAV1	0.928	-0.215	-1.143	4.91E-05	0.013
WAS	3.406	2.264	-1.141	2.59E-05	0.010
MRVI1	-1.099	-2.239	-1.141	0.0009362	0.045
CCR5	0.126	-1.006	-1.132	0.0006878	0.040
PCED1B-AS1	0.335	-0.796	-1.131	0.0003759	0.030
SLAMF8	2.168	1.044	-1.124	0.0001409	0.022
SH3RF3-AS1	1.030	-0.093	-1.124	0.0010015	0.046
CD74	8.556	7.433	-1.123	0.0006416	0.038
ICAM3	-2.287	-3.402	-1.116	0.000978	0.046

© 2021 Liu W et al. *JAMA Network Open.*

Gene symbol	Low risk (Mean of log 2(RPKM))	High risk (Mean of log 2(RPKM))	logFC	P Value	FDR
LGALS2	2.734	1.624	-1.110	0.0012233	0.049
IL2RG	1.720	0.614	-1.105	0.0005576	0.036
LINC01503	2.299	1.194	-1.105	0.000905	0.044
DAPP1	-1.687	-2.787	-1.100	0.0004191	0.032
LPAR5	1.557	0.458	-1.099	7.74E-05	0.017
CD53	4.714	3.620	-1.094	0.0002411	0.027
CD209	1.896	0.809	-1.087	0.000313	0.029
FCGR2A	2.034	0.950	-1.083	0.0001465	0.022
DACT1	3.124	2.056	-1.068	0.0002906	0.029
PDCD1LG2	0.972	-0.094	-1.065	0.0011073	0.047
C5AR1	3.219	2.161	-1.058	0.000313	0.029
PYCARD	3.403	2.346	-1.057	0.0004668	0.034
MS4A7	2.631	1.584	-1.047	0.0008748	0.044
ITGAM	1.163	0.119	-1.045	0.0002322	0.027
CD82	4.450	3.411	-1.039	8.75E-05	0.018
SIGLEC9	1.281	0.242	-1.038	0.0007629	0.041
PEAK3	-0.556	-1.587	-1.031	0.0001409	0.022
LGALS1	11.348	10.320	-1.028	0.001071	0.046
CEBPA	2.579	1.553	-1.026	0.000905	0.044
C1orf162	2.378	1.352	-1.026	0.0007371	0.040
PIPOX	-0.838	-1.855	-1.017	0.0005195	0.035
TLR8	-1.474	-2.491	-1.016	0.0011073	0.047
ADAP2	2.990	1.980	-1.010	0.0002411	0.027
PDE1B	-1.353	-2.364	-1.010	0.0005195	0.035
SERPINE1	3.824	2.816	-1.008	0.0011834	0.048
MAP3K5	1.083	0.080	-1.004	1.45E-05	0.008
TYMP	4.173	3.170	-1.003	0.0001204	0.020
CD37	1.870	0.870	-1.001	4.71E-05	0.013
TCF7	0.891	1.933	1.042	9.61E-06	0.008
PRTG	-1.946	-0.895	1.051	0.001071	0.046
MUC1	-0.120	0.947	1.067	0.0003247	0.029
SGMS2	2.534	3.623	1.089	0.0006416	0.038
PLCB4	0.435	1.541	1.106	0.0004191	0.032
ZDHHC23	-2.356	-1.209	1.146	0.0004345	0.033
B4GALNT1	0.446	1.635	1.189	0.0002906	0.029
DLX3	3.778	5.126	1.348	0.0002322	0.027
COL24A1	0.859	2.207	1.349	0.0003759	0.030
CGREF1	2.275	3.708	1.433	0.0006643	0.039
RHBDL2	3.330	4.800	1.470	0.0001157	0.020
COL13A1	1.417	2.901	1.484	4.71E-05	0.013

© 2021 Liu W et al. *JAMA Network Open.*

**Gene symbol**	**Low risk (Mean of log 2(RPKM))**	**High risk (Mean of log 2(RPKM))**	**logFC**	**P Value**	**FDR**
GALNT14	1.626	3.183	1.556	0.0003016	0.029
SP7	5.190	6.777	1.587	0.0001646	0.023
CYFIP2	-0.191	1.397	1.588	0.0006643	0.039
INSC	2.754	4.402	1.648	0.0003247	0.029

© 2021 Liu W et al. *JAMA Network Open.*

eTable 3. Top 10 Most Significant Terms in GO Enrichment Analyses

ID	Description	P.adjust	Count
Biological processes			
GO:0006954	inflammatory response	1.99×10^{-21}	180
GO:0050865	regulation of cell activation	2.94×10^{-20}	157
GO:0002694	regulation of leukocyte activation	3.80×10^{-20}	149
GO:0046649	lymphocyte activation	1.52×10^{-18}	175
GO:0002253	activation of immune response	5.15×10^{-17}	158
GO:0002250	adaptive immune response	5.02×10^{-16}	111
GO:0042110	T cell activation	2.51×10^{-15}	130
GO:0002696	positive regulation of leukocyte activation	4.35×10^{-15}	95
GO:0050867	positive regulation of cell activation	4.45×10^{-15}	97
GO:0051249	regulation of lymphocyte activation	2.13×10^{-14}	119
Molecular functions			
GO:0004888	transmembrane signaling receptor activity	5.71×10^{-9}	132
GO:0038187	pattern recognition receptor activity	9.80×10^{-6}	13
GO:0008329	signaling pattern recognition receptor activity	3.04×10^{-5}	12
GO:0005125	cytokine activity	7.24×10^{-5}	39
GO:0048018	receptor ligand activity	2.48×10^{-4}	73
GO:0004930	G protein-coupled receptor activity	5.72×10^{-4}	60
GO:0005126	cytokine receptor binding	6.29×10^{-4}	51
GO:0004896	cytokine receptor activity	6.29×10^{-4}	22
GO:0030545	receptor regulator activity	6.29×10^{-4}	74
GO:0016705	oxidoreductase activity, acting on paired donors, with incorporation or reduction of molecular oxygen	1.05×10^{-3}	34
Cellular components			
GO:0009897	external side of plasma membrane	1.04×10^{-9}	61
GO:0009986	cell surface	1.04×10^{-9}	143
GO:0098552	side of membrane	2.90×10^{-7}	85
GO:0045121	membrane raft	8.77×10^{-6}	76
GO:0098857	membrane microdomain	8.77×10^{-6}	76
GO:0098589	membrane region	1.65×10^{-5}	77
GO:0043235	receptor complex	2.58×10^{-4}	63
GO:0072562	blood microparticle	3.96×10^{-4}	28
GO:0030667	secretory granule membrane	4.55×10^{-4}	66
GO:0098802	plasma membrane receptor complex	4.55×10^{-4}	40

© 2021 Liu W et al. *JAMA Network Open.*

eFigure 1. Overall Survival According to the Risk Score in Patients With Osteosarcoma

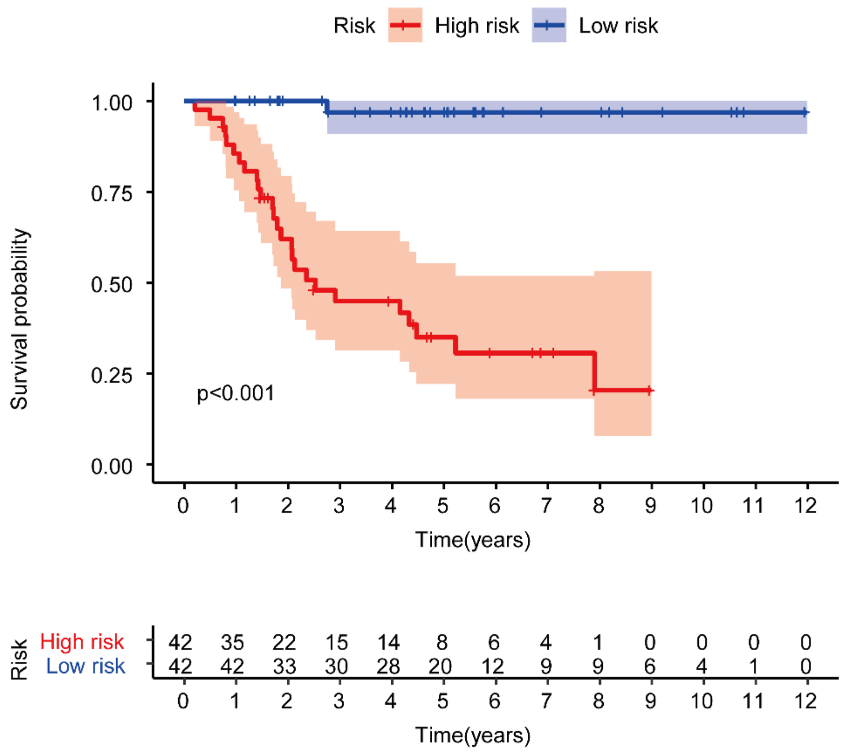

© 2021 Liu W et al. *JAMA Network Open.*

eFigure 2. Volcano Plot and Heatmap of DEGs Between Groups

A

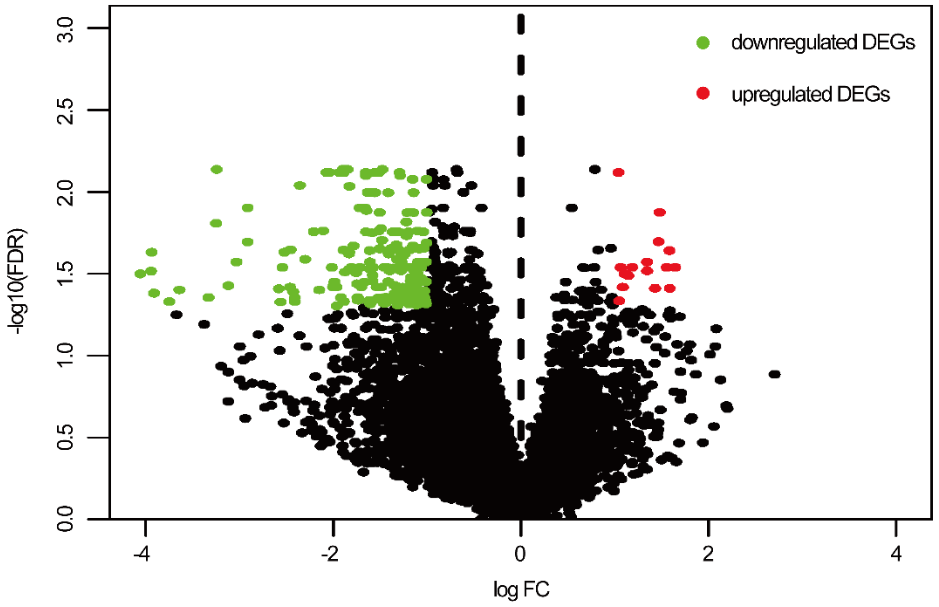

B

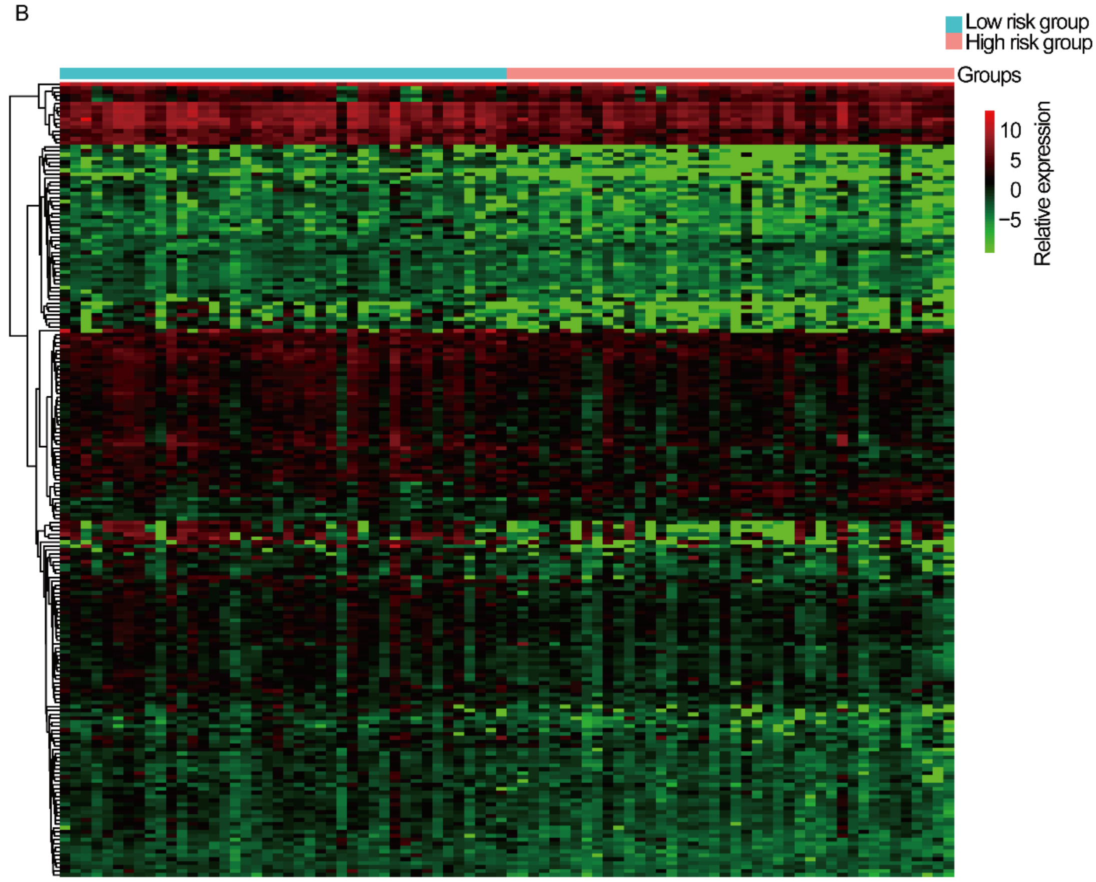

(A) Volcano plot. Red dots represent significantly upregulated DEGs in high risk group, while green dots represent significantly downregulated DEGs. (B) Heatmap. There were notable differences between the two groups according to their gene expression signatures.

© 2021 Liu W et al. *JAMA Network Open.*

eFigure 3. KEGG Pathway Enrichment Analyses: the Enriched KEGG Signaling Pathways From the DEGs

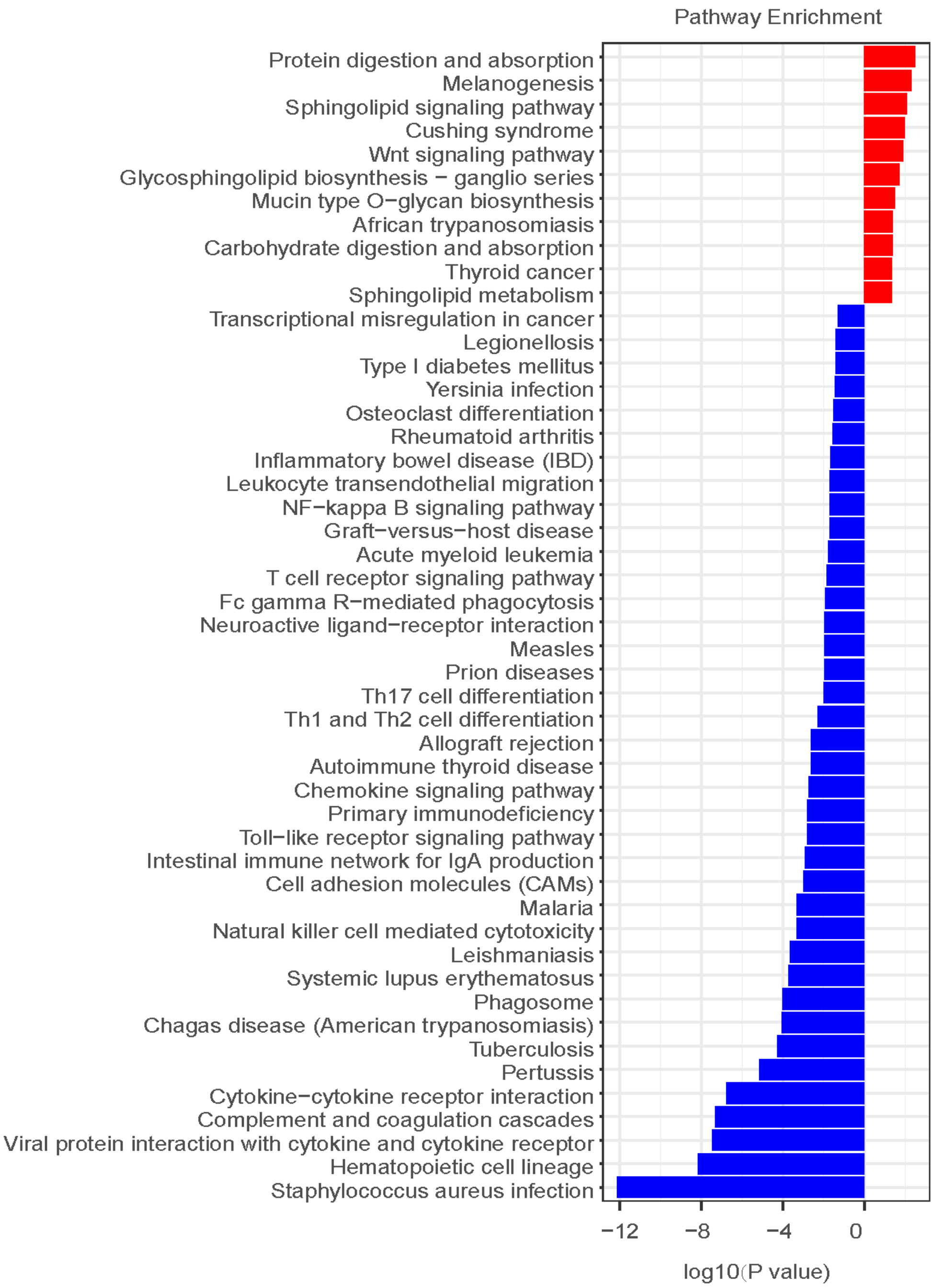

© 2021 Liu W et al. *JAMA Network Open.*

eFigure 4. TFs and IRGs Associations

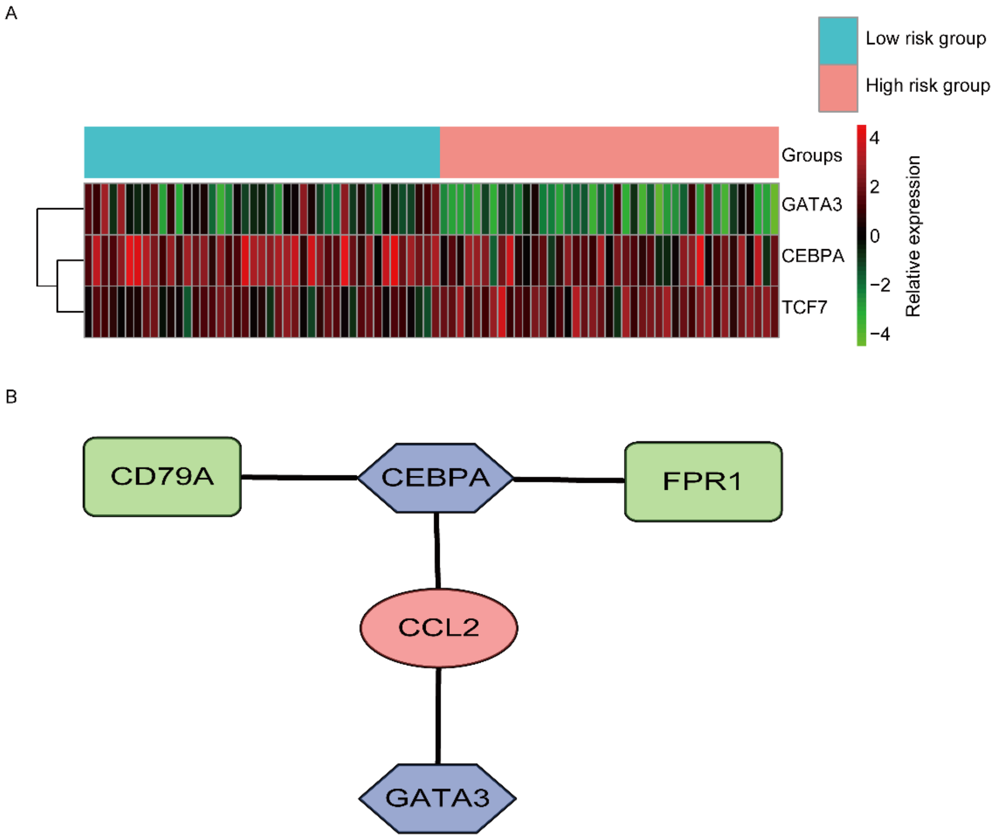

(A) Heatmap of differentially expressed TFs between two groups. (B) TFs-IRGs interaction network. Blue hexagon: TFs; green round rectangle: low risk IRGs; red ellipse: high risk IRGs.

© 2021 Liu W et al. *JAMA Network Open.*
